# Dynamic Relationships, Regional Differences, and Driving Mechanisms between Economic Development and Carbon Emissions from the Farming Industry: Empirical Evidence from Rural China

**DOI:** 10.3390/ijerph18052257

**Published:** 2021-02-25

**Authors:** Wenxin Liu, Ruifan Xu, Yue Deng, Weinan Lu, Boyang Zhou, Minjuan Zhao

**Affiliations:** 1 College of Economics and Management, Northwest A&F University, Yangling 712100, China; liuwenxin@nwafu.edu.cn (W.L.); xuruifan@nwafu.edu.cn (R.X.); dengyue@nwafu.edu.cn (Y.D.); luweinan@nwafu.edu.cn (W.L.); 2 College of International Relations, Xi’an International Studies University, Xi’an 710061, China; amado1988@163.com

**Keywords:** economic development, carbon emissions from planting industry, decoupling model, complete decomposition model, rural China

## Abstract

The coordinated development of the economy, resources, and environment is a key aspect of sustainable development. China’s rapid agricultural modernization has been accompanied by the continuous growth of rural economic aggregate and carbon emissions from the planting industry. However, the quantitative relationship between these two factors and its internal mechanism are not yet fully understood. In this paper, the Intergovernmental Panel on Climate Change (IPCC) method is used to calculate the carbon emissions of the planting industry in China from 1998–2019. Based on this, the Tapio decoupling analysis model was constructed to study the decoupling relationship between economic development and carbon emissions of the planting industry in China from 1998–2019 and the associated spatial and temporal evolution patterns. The effect of the complete decomposition model (without residuals), in terms of carbon emissions from the planting industry, on the process of economic development and its transmission mechanism are introduced. The results show that: (1) The carbon emissions of the planting industry in China increased with the economic development occurring from 1998–2005, where agricultural economic development was highly dependent on resource factors and the environment. The growth trend of carbon emissions of the planting industry slowed from 2006 to 2019, while economic development has gradually realized the decoupling of carbon emissions from the planting industry. (2) From 1998–2019, in Heilongjiang, Sichuan, and Hunan, the economic development was given priority, showing strong and negative decoupling with carbon emissions from farming. The economic development in most regions were given priority, showing strong decoupling with carbon emissions from farming. Up to 2019, decoupling was observed with a significant trend of spatial agglomeration. (3) Economic scale effects had a positive influence on the carbon emissions of the planting industry, while the technology effect and population effect had an inhibiting influence on the carbon emissions of the planting industry. The key policy implication of this paper is that improvement of the quality of economic development serves as the premise for the transformation of the economic development mode. It is necessary to reasonably regulate the economic growth rate and expansion scale, reduce resource consumption and pollutant emission technology, and to make full use of resources, in order to provide a basis for the formulation of reasonable emission reduction policies. An effective way to realize the sustainable development of the agricultural economy would be to improve the technical efficiency, control the population scale appropriately, and optimize the agricultural industrial structure.

## 1. Introduction

Climate change is a global issue of common concern to the international community. It is also one of the biggest, most extensive, and most far-reaching challenges mankind has faced [1]. With the rapid advancement of economic globalization, human consumption of global natural resources has reached an unprecedented level [2]. The agricultural economic development is highly associated with climatic change, and also causes dangerous activity. Carbon emissions are the main source of climate variability. The increasing severity of extreme weather means that farmers often face erratic rainfall, pests, and natural disasters. [3,4]. As the main component of greenhouse gases, the annual growth of carbon cannot be separated from human deforestation and use, along with the burning of a large amount of fossil fuels [5,6]. Therefore, countries worldwide have sought to reduce carbon emissions, as the focus of their own emission reduction work. According to the Intergovernmental Panel on Climate Change (IPCC), agricultural activities in all countries contribute 13.5% to global carbon emissions [7]; thus, carbon emissions caused by agricultural production have become an important source of the increase in global greenhouse gases [8]. Climate change usually shortens the growth cycle of food crops, and reduces the average production, which further impact on economic development [4]. China is an agricultural powerhouse, with farming systems responsible for about 16% of the country’s total greenhouse gas emissions [9,10]. In 2018, China’s carbon emissions caused by planting production reached 96.71 million tons, a rise of 51% compared with 1997 [11]. In order for China to meet its commitments under the Paris Agreement, it must reduce carbon emissions originating from farming activities. China is a large country, however, and differences between regions, such as water, soil, air temperature, and sun exposure, leading to obvious basic agricultural production factor endowment differences, which may lead to the agricultural development level and carbon emission levels differing clearly between the regions of China [12,13,14,15]. With the development of the economy and the industrial chain of regional transfer and diffusion of technology, dynamic changes cannot be made, in terms of China’s regional planting industry development level [16,17]. With the rapid development of agricultural modernization in China, a large percentage of the rural non-agricultural population is expected to increase crop production through the use of alternative labor, such as chemical fertilizers, pesticides, and agricultural machinery, which have greater resource demands; such resource consumption will eventually return, in the form of waste, into the environment [18,19,20]. Furthermore, a huge increase in the consumption of carbon sources is also expected, due to improved carbon-based planting technology. It has long been believed that the objective of materialization can be achieved by improving technical efficiency, thus alleviating the problem of resource shortages in China. However, despite the obvious improvement in technical efficiency since the Industrial Revolution, environmental pollutant emissions are still increasing in most countries [21]. The reason for this is that the improvement of efficiency is relative to other elements (e.g., cheap resources) and, through more rapid economic growth, it can also produce new resource consumption requirements at the same time, thus becoming partially offset by saving resources and emitting more carbon emissions, which produces a “rebound effect” [22]. Obviously, this counteracts the development requirements of low-carbon and green agriculture in China.

Research on the relationship between economic growth and carbon emissions from the planting industry has become a hot topic. Some scholars believe that carbon emissions from the planting industry, mediated by the process of economic growth, are the main cause of rural environmental pollution [23], where the severity of carbon emissions from the planting industry may exceed the crisis caused by reduced water use and reduced arable land [24]. Some scholars believe that the extensive mode of agricultural growth is still the main mode of agricultural economic growth in China—even if the agricultural modernization level is no longer decreasing and may continue to grow [25]—thus stressing the need to reduce carbon emissions and to optimize the combination of planting industry carbon emissions and economic growth. Supporting and constraining effects have been proposed, which do not necessarily imply a positive relationship [26]. The improvement of economic growth may inhibit planting carbon emissions [27,28]; even if the agricultural economy is growing at a rapid rate, carbon emissions from the planting industry may grow slowly or negatively [29]. At present, China’s rural areas are still in the process of agricultural modernization and rapid economic development. In order to achieve the established emission reduction targets, we must reduce the dependence of economic growth on resource consumption. Uncontrolled carbon emissions from the planting industry will act on economic growth in two ways: one is related to the function of the natural environment to absorb and deposit wastes. By consuming the natural environment, economic activity units can increase their output level on the premise of given other input factors, thus bringing about an overall positive impact on economic growth [30,31]. However, carbon emissions from the planting industry will significantly reduce the attractiveness of rural areas, thus restricting the effectiveness of increasing returns to scale and agglomeration effects in rural areas, ultimately slowing down economic development. In other words, carbon emissions from the planting industry can affect economic development [32]. The ultimate impact of such unregulated carbon emissions on economic development depends on the relative change of the positive and negative effects. In order to contain the uncertain and extensive growth mode, it is necessary to “decouple” the pressure of resources and the environment from economic growth, and to effectively ensure energy saving, consumption reduction, and environmental protection [33].

Therefore, the causal relationship between economic growth and the carbon emissions of the planting industry is uncertain. Focusing on the above issues, this study attempts to make progress in the following aspects. By judging the causal relationship between China’s economic development and carbon emissions from the planting industry, further clarifying the degree of mutual influence between economic development and carbon emissions from planting industry, we ask: in the stage of rapid agricultural modernization in China, is the contribution of economic development to the carbon emissions of the planting industry positive or negative? What is the extent of the impact? Is the law of variation consistent at different spatial and temporal scales in China? Furthermore, will economic development significantly control carbon emissions from farming, and what will be the impact of this? Therefore, based on the IPCC method, we estimated the carbon emissions of the planting industry in China. On this basis, the decoupling model was used to explore the relationship between economic development and carbon emissions of the planting industry. Furthermore, we developed a complete decomposition model of carbon emissions, a quantitative measure of the effect of economic development on the planting industry-driven carbon emissions in China’s 31 provincial administrative regions, up to the provincial level, in an attempt to determine the key factors influencing planting carbon emissions, as well as to explore the agricultural economy and the development of a coupling law for carbon emissions, in order to develop and implement environmental and efficient resource utilization policies and to provide a scientific basis for such policies to improve the quality of economic operations.

## 2. Literature Review and Research Innovation

In 2008, McKinsey made it clear, in the “Carbon Productivity Challenge: Curbing Global Change and Sustaining Economic Growth”, that any successful climate change mitigation technology must support two goals—to stabilize greenhouse gas levels in the atmosphere and to sustain economic growth [34]. Therefore, the relationship between economic growth and carbon dioxide emissions has been a long-standing focus of academic attention. The importance of low-carbon and green development has become a consensus worldwide. Scholars from all countries have also started to calculate carbon emissions, considering such fields as energy consumption [35], construction [36], and agriculture [37]. Other scholars have analyzed the relationship between climate change and economic development [4,38]. With the increase in agricultural carbon emissions, the issue of agricultural carbon emissions has attracted the attention of researchers worldwide, especially those in developing countries. Carbon emissions from the planting industry are an important part of agricultural carbon emissions. Carbon emissions from the planting industry mainly come from the demand for fossil fuel energy in agricultural production, as well as the input of production materials such as chemical fertilizers, pesticides, and agricultural films, produced in the pursuit of high yields [39,40]. Research on the influencing factors of carbon emissions from the planting industry has long been the focus of scholars at home and abroad. However, due to the numerous and complex factors affecting carbon emissions, these scholars have conducted research and analyses from different perspectives and using various methods, mainly including the following three aspects: (1) Study of carbon emissions measurement and driving factors for the planting industry. Research on the carbon emissions of the planting industry mainly focuses on the measurement of carbon emissions and the associated driving factors [41]. It has been found that the carbon emissions caused by the consumption of agricultural materials in the production processes of the planting industry show an overall growth trend, among which the carbon emissions caused by the application of chemical fertilizers contributed the most [42]. From the perspective of driving factors, the impact of planting industry development on carbon emissions had an inverted “U” shape, while the scale of agricultural labor force, industrial structure, and production efficiency of the planting industry could inhibit carbon emissions. (2) Discussing the overall relationship between economic growth and carbon emissions, such as co-integration and Environmental Kuznets Curve (EKC) curve relationships. The main concepts of EKC are as follows: at a low level of economic development, carbon emissions and other pollutants will increase along with economic growth. After a certain inflection point, economic growth will be accompanied by a decline in emissions and other pollutants. The research results relating to the EKC have been controversial [43]. Scholars Carson and Saboori concluded that the EKC hypothesis was established after an empirical study on the data of the United States and Malaysia, respectively. Richmond and Kaufman, however, found no “inverted U-shaped” relationship between economic growth and carbon emissions. In the study of EKC curves, Chinese scholars have found that the relationship between the two is an “inverted U-shaped” in most provinces, but are also “inverted N-shaped” or “positive U-shaped” in a small number of provinces and cities, which may be due to different selection indices and regional differences in the eastern, central, and western regions. Some scholars believe that industrial structure or industrial upgrading have impacts on the EKC curve [44]. For example, Wang et al. [45] found that the relationship between economic growth and air pollutant emissions was not completely consistent with the traditional “inverted U-shaped” EKC curve, by utilizing a spatial Durbin lag model and a semi-parametric spatial lag model. (3) Current studies on the spatial heterogeneity of carbon emissions in China have mostly focused on the carbon dioxide emissions of cities at the provincial level [46,47]. For example, Wei [46] studied the relationship between economic growth and carbon emissions in south and north China using a grey prediction mode. Zhang [48] found that the correlations between carbon emission technologies and land-use benefits in Zhengzhou were significantly different for different industries, where the decoupling state fluctuated significantly. From 2012 to 2015, there was mainly negative and strong decoupling. This was mainly due to the fact that China’s energy consumption list data are mostly concentrated at the national, provincial, and big-city levels, with a lack of a relatively complete energy balance sheets for carbon emissions from the planting industry over a long period of time. This is also one of the reasons why most studies on carbon emissions of the planting industry in China have focused on case studies. However, because agricultural machinery, pesticide, fertilizer, and other emissions monitoring, reporting, and verification are constantly improved and better estimation methods are developed, data with different spatial data quality at a provincial scale of planting industry carbon emissions have become available, facilitating the spatial heterogeneity of research into the effect of planting on carbon emissions at larger scales [49].

From the above literature review, it can be seen that most of the existing relevant studies have mainly focused on a local area and did not carry out analysis or comparison at a national scale. Therefore, it is of certain practical significance to study the spatiotemporal evolution characteristics of the relationship between economic development and carbon emissions of the planting industry in China from the perspective of the whole country. The research innovation of this paper is mainly reflected in the following three aspects. Firstly, this paper utilizes data of 31 provinces from 1998 to 2019. It is relatively rare to use such a long time-series covering almost all provinces in China, in order to study the carbon emissions and economic development of the planting industry. Secondly, unlike the EKC relationship between economic development and carbon emissions in most of the literature, this paper systematically investigates the coupling and coordination relationship between economic development and carbon emissions from the perspective of decoupling. Thirdly, this paper systematically studies the decomposition of the transmission mechanism of carbon emissions of the planting industry caused by economic development, in order to comprehensively investigate the main factors affecting carbon emissions of the planting industry in the process of economic development.

## 3. Research Methods and Data Sources

### 3.1. Carbon Emissions Measurement Model for the Planting Industry

From the Intergovernmental Panel on Climate Change (IPCC) 2006 national greenhouse gas inventories guide-related practices, we used the carbon emissions factor method to calculate those which were associated with planting, namely the usage of different carbon sources and their corresponding emissions factors multiplied by the different emissions sources, followed by the carbon emissions of different carbon accumulation factors, to obtain the overall planting carbon emissions [50,51]. The specific formula is:

(1) $$c=\sum c_{i}=\sum e_{i}\times \delta_{i}$$

where c represents the total carbon emissions of the planting industry, $c_{i}\;$represents the carbon emissions of type i carbon sources of the planting industry, $e_{i}$ represents the usage amount of type i carbon sources, and$\;\delta_{i}$ represents the emissions coefficient of type i carbon sources. According to the recommended practices of the IPCC (2006, 2007 Guidelines for Compilation of Provincial Greenhouse Gas Inventory, Trial), issued by the National Development and Reform Commission of China in 2011, and the research results of relevant scholars [4,5,6,7,8], we divided the major carbon sources of the planting industry into chemical fertilizers, pesticides, agricultural films, agricultural diesel, agricultural seeding, and agricultural irrigation. The carbon emission coefficients of six types of carbon sources and their sources are shown in Table 1.

### 3.2. Research Methods of Decoupling Effect

“Decoupling” comes from the field of physics, indicating that two or more physical quantities which previously had a response relationship no longer have a relationship [52]. A “decoupling index” is often used to reflect the degree of non-synchronous change between resource consumption and economic growth, aiming to reflect the uncertain relationships between resource consumption, environmental pressure, and economic growth. At present, there are two main decoupling research models. The first is the decoupling factor model proposed by the Organization for Economic Co-operation and Development (OECD), based on the initial and final values of the period [53]. The second type is the decoupling index model proposed by Tapio, based on the change of growth elasticity [54]. The Tapio model takes the relative quantity change and the total quantity change into comprehensive consideration, adopting the elastic analysis method with time scale to reflect the decoupling relationships between variables. This method overcomes the difficulty of the OECD decoupling model in selecting the base period and further improves the objectivity and accuracy of the decoupling measure. The Tapio decoupling model was developed on the basis of the OECD decoupling model. The concept of decoupling was redefined by the “elasticity of decoupling”, and the types of decoupling were subdivided to construct the decoupling model, as follows [55]:

(2) $$\varepsilon =\frac{\Delta E∕E}{\Delta G∕G}$$

where ε is the elasticity of decoupling; E is the carbon emissions of the planting industry (CEP); and G is the agricultural gross domestic product (AED). According to the different elasticity values, the decoupling can be divided into six categories: strong decoupling, weak decoupling, recessionary decoupling, extended negative decoupling, weak negative decoupling, and strong negative decoupling. Strong decoupling means the more ideal the decoupling of resources, environmental pressure, and economic growth, the better the coordinated development state of the resources, environment, and economy. Strong negative decoupling means the more nonideal decoupling of resources, environment pressure, and economic growth, the worse the coordinated development state of the resources, environment, and economy. The specific grading is shown in Table 2.

### 3.3. Complete Decomposition Model Without Residual

A complete decomposition model is a factor decomposition method that completely eliminates the influence of residuals, which has been applied, to a certain extent, in the fields of energy, environmental ecology, and water resources [22]. Based on the factor-decomposition model without residual items, we constructed a complete decomposition model of carbon emission changes driven by economic development for the planting industry and decomposed the residual items, according to the “jointly caused and equally distributed” principle [29,56]. The following is a simple derivation of the model.

The change of carbon emissions from the planting industry is regarded as being influenced by population effects, economic scale effects, and technology effects. Therefore, we further improved the model as follows:

(3) I=P·A·T

In the equation, I represents the carbon emissions of the planting industry generated in the process of economic development; P denotes the total population; A is GDP per capita, representing economic scale; and T is technology, which can be expressed by the carbon emissions of the planting industry per unit GDP, i.e., T=I∕GDP. The equation can be expressed as:

(4) $$I=P\cdot \frac{GDP}{P}\cdot \frac{I}{GDP}=P\cdot A\cdot T$$

That is, the I variables are determined by the factors P, A, and T. Within the time period $\left[0,\;t\right]$, the variation of the variable can be calculated, according to Equation (4):

(5) $$\Delta I=I^{t}-I^{0}=P^{t}\cdot A^{t}\cdot T^{t}-P^{0}\cdot A^{0}\cdot T^{0}=\Delta P\cdot A^{0}\cdot T^{0}+\Delta A\cdot P^{0}\cdot T^{0}+\Delta T\cdot P^{0}\cdot \;A^{0}+\Delta P\cdot \Delta T\cdot A^{0}+\Delta P\cdot \Delta A\cdot T^{0}+\Delta A\cdot \Delta T\cdot P^{0}+\Delta P\cdot \Delta A\cdot \Delta T$$

where $\Delta P\cdot A^{0}\cdot T^{0}$, $\Delta A\cdot P^{0}\cdot T^{0},\;\Delta T\cdot P^{0}\cdot A^{0}$ are the contributions of the respective changes of factors P, A, T to the total change of variables; $\Delta P\cdot \Delta A\cdot T^{0}$ is the contribution of the change of factor P, A synthesis to the total change of the variable; ∆*P*∙∆*T*∙*A*^0^ is the contribution of the change of factor *P*,*T* synthesis to the total change of the variable; ∆*A*∙∆*T*∙*P*^0^ is the contribution of the change of factor A, T synthesis to the total change of the variable; and ∆*P*∙∆*A*∙∆*T* is the residual amount in the fully decomposed model. According to the principle of equal distribution, the complete decomposition model of the system consisting of three factors is as follows:

(6) $$P_{effect}=\Delta P\cdot A^{0}\cdot T^{0}+\frac{1}{2}\Delta P\left(A^{0}\cdot \Delta T+T^{0}\cdot \Delta A\right)+\frac{1}{3}\Delta P\cdot \Delta A\cdot \Delta T$$

(7) $$A_{effect}=\Delta A\cdot P^{0}\cdot T^{0}+\frac{1}{2}\Delta A\left(P^{0}\cdot \Delta T+T^{0}\cdot \Delta P\right)+\frac{1}{3}\Delta P\cdot \Delta A\cdot \Delta T$$

(8) $$T_{effect}=\Delta T\cdot P^{0}\cdot A^{0}+\frac{1}{2}\Delta T\left(P^{0}\cdot \Delta A+A^{0}\cdot \Delta P\right)+\frac{1}{3}\Delta P\cdot \Delta A\cdot \Delta T$$

It can be seen, from the formula, that the carbon emissions of the planting industry are mainly affected by the $P_{effect}$,$\;A_{effect}$, and $T_{effect}$. $P^{0}$, $A^{0}$, $T^{0}$ are expressed by the total population, per capita GDP, and carbon emissions degree of the planting industry per unit GDP, respectively; in more detail, ΔP, ΔA, ΔT are the change in total population, the change amount of per capita GDP, and the change amount of carbon emissions per unit GDP from the planting industry at the end of the period relative to the base period, respectively. The change in carbon emissions from the planting industry is regarded as the result of the combined action of these three factors. The calculation method and principle of each variable are the same as in Equations (3)–(8).

### 3.4. Data Sources

The total carbon emissions of the planting industry were mainly calculated according to the formula published by the IPCC. If individual values in the statistical yearbook were missing, the mean value of the sum of values of adjacent years was adopted. It is important to note that China’s growth had been reported in nominal GDP, which did not take inflation into account. Based on this data background, in order to ensure the objectivity and rigor of the research, the agricultural GDP adopted in this paper was the actual agricultural GDP calculated from the agricultural GDP published in 1978 (as the base period). At the same time, due to imperfect management systems, there was a lack of a unified summary and compilation of data on chemical fertilizers and pesticides in China for a quite long period of time, which was not officially released until 1997. Based on the above two factors, we selected data from 1998 to 2018 to conduct an empirical analysis on the economic growth and carbon emissions of the planting industry in the process of agricultural modernization. Data sources were the China Rural Statistics Bulletin, China Environmental Statistics Yearbook, and China Statistical Yearbook, unless otherwise stated. In the end, for a more comprehensive evaluation of agricultural modernization, we referred to the China Environmental Statistics Yearbook, selecting the agricultural mechanization rate index as a proxy variable for agricultural modernization, using agricultural modernization variables as a tool to assess the planting industry carbon emissions and economic growth variable; thus, we could fully explore the effects of carbon emissions of the planting industry on and economic development under a more comprehensive frame.

## 4. The Dynamic Relationship Between Economic Development and Carbon Emissions of the Planting Industry

### 4.1. Overall Description of Economic Development and Carbon Emissions of the Planting Industry

**Figure 1:**
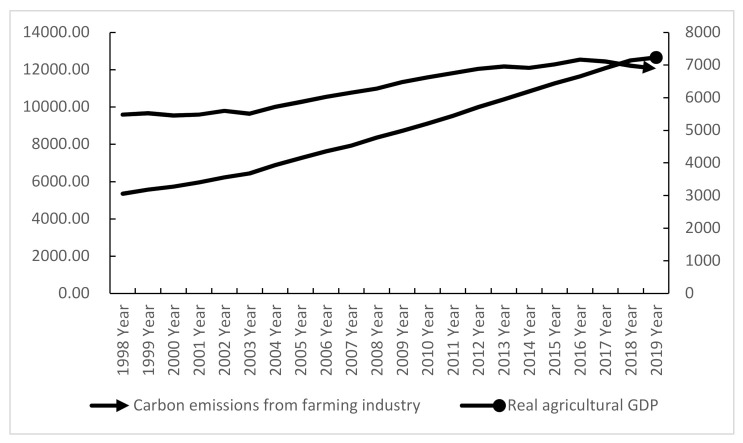
The trend of economic development and carbon emissions from planting.

Taking 1978 as the base period, China’s rural GDP and carbon emissions of the planting industry were calculated from 1998 to 2019; the change trend is shown in Figure 1. In general, the variation trend of China’s rural GDP and carbon emissions of the planting industry from 1998–2019 was basically the same, where the carbon emissions of the planting industry in China showed a general annual trend of increasing with an increase in GDP, from 95.8439 million tons in 1998 to 120.5912 million tons in 2019. From 1998–2003, the growth trend of rural GDP and carbon emission of the planting industry was flat. During this period, China’s rural economy was in the development mode of “high input and high consumption”, being strongly dependent on the planting industry. During this period of China’s rural economic development, the traditional extensive mode was in the dominant position and the rural economy was mainly based on the planting industry, while the development of animal husbandry and rural non-agricultural industry was relatively slow, which greatly restricted the overall benefits of the rural economy. From 2004 to 2016, both rural economic development and carbon emissions of the planting industry showed a rapid growth trend. Among them, the growth rate of the rural economy was faster than that of carbon emissions of the planting industry, where the change of carbon emissions of the planting industry tended to be stable. This was mainly because, after joining the World Trade Organization (WTO) and under the background of the party’s rural policy release, the provinces relied on resource advantages to vigorously develop their rural industry, optimizing the rural industrial structure, promoting the agricultural modernization level, planting, animal husbandry, aquaculture, and the comprehensive development of non-agricultural industries and, to a certain extent, eliminating the disadvantages of the urban–rural dual structure, such as education and healthcare; thus, the new rural construction formed an important context of the national rural policy. However, with the development of the rural economy, the consumption of agricultural factor resources increased rapidly, while the pressure of environmental pollution increased slowly. From 2017 to 2019, the rural economy still maintained its rapid increasing trend. It is worth noting that, considering China’s farming carbon emissions in 2016, there was an obvious turning point—it reached its highest level since the 21st century, otherwise being in decline. In April 2016, China joined the Paris agreement and the whole country moved towards establishing a low-carbon green society. Environmental protection was strengthened throughout the provinces during this period, with many fertilizers and pesticides obtaining a certain degree of limitation; as a result, the number of chemical fertilizers used has fallen sharply. With the in-depth promotion of agricultural modernization and rural revitalization policies, the reduction in agricultural water, soil, fertilizer, and other resources has achieved certain results, leading to a downward trend in the total carbon emissions from the planting industry.

### 4.2. Granger Causality Test of Economic Development and Carbon Emissions of the Planting Industry

From Figure 1, it is not difficult to see that the agricultural economic development (AED) of economic development and the carbon emissions from planting (CEP) had a relatively consistent overall development trend. Therefore, a co-integration analysis of the two was carried out. Due to the unit root problem of many data, the logarithm is usually taken first, and then the difference method is used. The logarithmic treatments of AED and CEP are denoted as LAED and LCEP, respectively. There are many methods for unit root testing, such as the Phillipsand Perron (PP), Augmented Dickey–Fuller (ADF), and Kwiatkowski-Phillips-Schmidt-Shin (KPSS) tests, among others. In this paper, the commonly used ADF test was carried out to determine the order of a single integral (Table 3).

The specific test results are shown in Table 3, where i represents the first-order difference; DW represents the Durbin–Watson test value; and ADF represents the unit root test value. It can be seen that the *t*-statistic values of LAED and LCEP were −2.579 and −2.469, respectively, both larger than the critical value of −3.742, at the 5% significance level, indicating that the level sequences of LAED and LCEP were non-stationary. However, their first-order difference sequences iLAED and iLCEP were determined to be stationary after the unit root test, where the ADF statistics of −1.976 and −1.432 were both smaller than the critical values of −1.085 and −1.369, respectively, with a significance level of 5%. Therefore, LAED and LCEP are first-order single integral time series I. Therefore, it can be considered that they may be affected by some common factors and show the same trend in time; that is, there may be a stable co-integration relationship. The test data of the regression showed that there was an obvious co-integration relationship between LAEG and LCEP at the critical value of 5%. The Granger causality test results between economic development and carbon emissions from the planting industry are shown in Table 4.

The test results showed that there was an obvious one-way causal relationship between economic development and carbon emissions from the planting industry. The increase or decrease in economic aggregation inevitably leads to an increase or decrease in carbon emissions of planting industry. Based on this, a regression equation was established, as follows:

(9) LZCP=1.459+0.286×LAEG

(10) $$Adjusted\;R^{2}=0.756\;\;\;\;\;\;DW=0.875\;\;\;\;Prob\;\left(F-statistic\right)=0.0036$$

The DW value in the model was small, which indicated that the error term had an autocorrelation problem; the first-order auto regressive $AR\;\left(1\right)$ was added to the original regression equation model of planting industry carbon emissions to the rural economic aggregate, and the generalized difference regression results were obtained. According to the results of the generalized differential regression analysis, the adjusted *R*^2^ increased from 0.756 to 0.832, the DW statistic increased from 0.875 to 1.834, and all statistics passed the significance test. Thus, the generalized differential regression model of LCEP on the LAED was obtained as:

(11) $$LCEP=1.325+0.435LAED+\left[AR\left(1\right)=0.453\right]$$

According to the results of the generalized differential regression analysis, if the LAEG increased by 1%, the LCPE increased by about 0.45%. Therefore, it can be stated that the carbon emissions of the planting industry in China increase with the economic development.

### 4.3. The Decoupling Relationship Between Economic Development and Carbon Emissions of the Planting Industry

**Figure 2:**
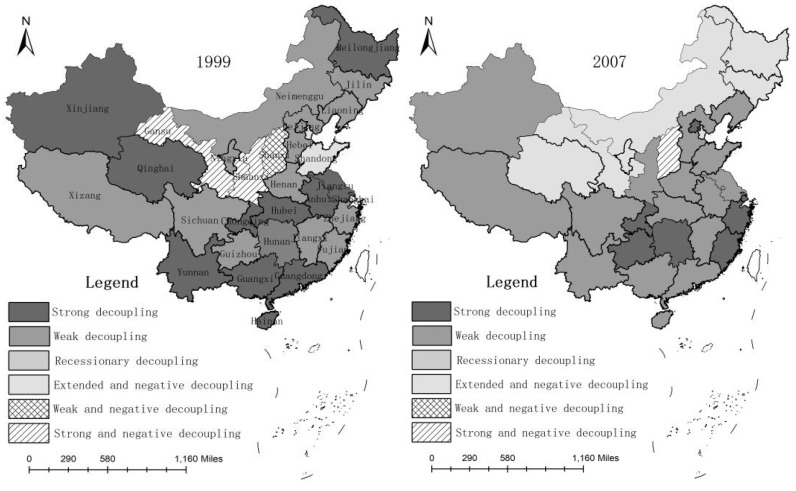

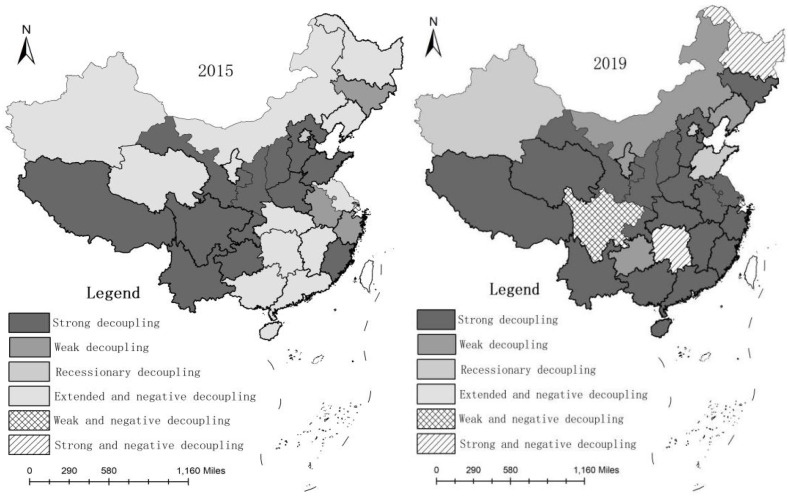
The temporal and spatial difference of decoupling relationship.

In combination with the criteria of decoupling degree and the calculation results of the decoupling elasticity index between agricultural economic development and carbon emissions from the planting industry (Table 5), GIS spatial analysis technology was used to analyze the spatial evolution pattern of the decoupling degree between agricultural economic growth and carbon emissions from the planting industry in 31 provinces of China, as shown in Figure 2.

In 1999, the regional distribution of strong and weak decoupling between agricultural economic development and carbon emissions from the planting industry was relatively disperse, where the degree of decoupling had great regional differences. Beijing, Hebei, Inner Mongolia, Liaoning, Jilin, Zhejiang, Fujian, Jiangxi, Henan, Hunan, Sichuan, Guizhou, Tibet, and the agricultural economic development of Ningxia and planting were given priority, with weak decoupling between carbon emissions; the agricultural economic development of Tianjin, Shanghai, Shandong, and planting were given priority, with expansion causing negative decoupling between carbon emissions: that is, when these provinces produce rural economic growth, planting carbon emissions rise. This period gave priority to food crops (traditional agriculture), with a highly extensive growth mode. The development of animal husbandry, tourism agriculture, and processing agriculture was limited to a few developed regions, while the pressure on the environment was increasing. Due to the limitations of technology and equipment, the production scale was small, the product category was singular, and the agricultural industrial structure level was low. In Shaanxi and Gansu, there was a strong negative decoupling between the development of the agricultural economy and carbon emissions of the planting industry. These two provinces experienced economic recession, while carbon emissions from the planting industry increased significantly. These provinces are in the west, which was generally poor and arid. Before the national western development policy in 2000, confined to the two province’s own economic basis and climate conditions, the mechanization level was low and the agricultural economy of the two provinces showed a tendency of slow development, or even decline; however, in the process of development of the planting industry, the widespread use of chemical fertilizers, to a certain extent, made up for the shortage of water resources affecting food production, thus making the carbon emissions of the planting industry to seem higher.

In 2007, the elastic characteristics of carbon emissions from economic development and the planting industry were concentrated within weak decoupling and expanding negative decoupling, while strong decoupling significantly decreased. During this period, the decoupling indices of Heilongjiang, Jilin, Inner Mongolia, Shanxi, and Qinghai fluctuated greatly, decreasing by two degrees. The relationship between economic development and carbon emissions from the planting industry showed a significant negative regression. Heilongjiang, Jilin, and Inner Mongolia, as major grain-producing areas in China, conducting food production towards national fulfilment, the development of the agricultural economy was more dependent on national policy adjustment and non-market factors. Crop productivity was not high in this area, and the lack of support through agricultural manufacturing endowments was limited by the agricultural economy, which was in a state of slow development. On the other hand, in order to enrich the soil, chemical fertilizers and pesticides were used extensively, and agricultural mechanization became relatively widespread in the two provinces. The use of diesel oil here was among the highest in China. Carbon emissions from the planting industry experienced a rapid development trend. Although Shanxi well-deserves the name of “small coarse grain kingdom”, the reality that it is a small agricultural province cannot be changed. The low industrialization level was the “bottleneck” that restricted the development of characteristic agriculture in Shanxi Province. The development level of modern agriculture in Shanxi Province was still relatively low and there were many problems, such as small scale of leading industry, low degree of regional concentration, and weak agricultural economic development. Shanxi’s agriculture environmental problem is more special, due to its huge carbon emissions in industrial production; Shanxi is China’s largest coal province. In the province, farmland atmospheric sulfur dioxide, dust, and particulate matter pollution have presented a worsening trend, posing a serious threat to the development of agricultural production space, with serious agricultural irrigation water pollution and chemical oxygen demand (COD) up to 10 (or even 100) times higher than the national standard. Coal production and transportation have led to great pollution of the Shanxi agricultural ecological environment, such that the economy and development of the elastic characteristics of carbon emissions presented a strong negative decoupling state. Qinghai’s agricultural inputs and outputs have experienced a serious non-coordination phenomenon, which has become obvious with the continuous development of the modern economy, leading to a shortage of investment, over-extensive agricultural facilities, and an agricultural development lag phenomenon. Qinghai’s agricultural service system was not sufficient; furthermore, the cultivation of agricultural product structure varieties also exhibited a serious unreasonable phenomenon, which had a significantly negative impact on the development of the agricultural economy, mainly relating to irrigation in the process of developing agriculture; the river pollution was serious, with 300 thousand tons of industrial waste water discharged daily into the main agricultural irrigation source, leading to severe crop pollution. At the same time, because China’s main pastoral areas are in Qinghai province, high amounts of chemical fertilizers and pesticides were used in the process of grassland development, playing a huge role in planting carbon emissions.

In 2015, the decoupling indices of Hunan, Hubei, Jiangxi, Guangdong, Guangxi, Hainan, Liaoning, Jiangsu, and other provinces decreased, to different degrees, showing a state of expansion of negative decoupling. These areas showed obvious spatial characteristics. Affected by the international financial crisis and the influence of lag effects, the agricultural economies of Guangdong, Guangxi, Hainan, Liaoning, and coastal provinces, such as Jiangsu, presented recession or slow growth trends, while secondary and tertiary industries presented rapid growth, such that resource and environmental pressure increased significantly. Hunan, Hubei, and Jiangxi still belonged to the traditional agricultural development model, such that the agricultural structure was still the typical urban–rural dual structure, thus hindering the free flow of production factors. On the other hand, the lack of investment in agricultural infrastructure and the weak ability of disaster resistance and mitigation restricted the development of the agricultural economy. These regions developed earlier and faster in China, with more developed township enterprises, rapid population growth, and a large amount of wastewater discharge, leading to an increase in agricultural pollution. In Guangdong Province, for example, the amount of chemical fertilizer applied in 2015 increased by 1.5-fold, compared with that in 1999. Due to the influence of natural conditions and unreasonable land use, some cultivated land has produced serious non-point source pollution; consequently, their resources and environment are under great pressure.

In 2019, on the whole, the number of provinces with strong and weak decoupling at the regional level increased rapidly. Strong decoupling showed a significant spatial agglomeration trend. Except for Heilongjiang, Sichuan, and Hunan, the gap of regional decoupling degree gradually narrowed. These provinces relied on national policy and their industrial base to speed up the transformation of the pattern of economic development, vigorously developing processing industries for fresh water aquaculture, agribusiness, sightseeing agriculture, and other fields, creating high value-added agricultural products through deep processing, realizing the efficient use of agricultural resources, thus facilitating agricultural economic growth while planting carbon pressure showed a trend of slowing or declining. Heilongjiang, Hunan, and Sichuan, as traditional agricultural provinces, showed strong or weak negative decoupling states, indicating that, in these three provinces, agricultural economic development was relatively slow and even reversed, while planting resource consumption was still in the high consumption, high energy consumption, and low efficiency mode, causing agricultural economic growth and a planting carbon pressure surge. The common characteristic of these three provinces is the regional water shortage problem. The contradiction between the rapid industrialization, urbanization, and agriculture competition for resources became increasingly fierce, compared to the rest of the country; crop diseases and insect pests were multiple, frequent, and often retransmitted, resulting in farmers relying excessively on fertilizer and chemical inputs such as pesticides, herbicides, pesticides, and agricultural films, leading to an agricultural ecological resource environmental load increase. Ecological and environmental problems related to agriculture have become increasingly prominent in these provinces, such as the degradation of cultivated land, aggravation of environmental pollution, and heavy metal pollution.

### 4.4. Analysis on the Rebound Effect of Carbon Emissions of the Planting Industry

According to the rebound effect model constructed by Equations (3)–(8), the specific impact degrees of population growth, economic scale, and technical efficiency on carbon emissions of the planting industry in the process of economic development in 2019 was calculated, as shown in Appendix B. The population effect reflected the demand on the planting industry generated by population growth, which led to a change in carbon emissions from the planting industry. The scale effect reflected the demand on the planting industry caused by the expansion of the agricultural economy, which led to a change in the carbon emissions of the planting industry. Technology effects reflect technological advances and availability.

**Figure 3:**
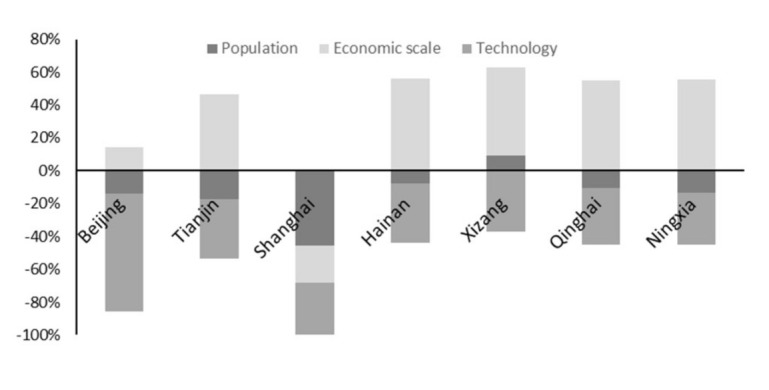

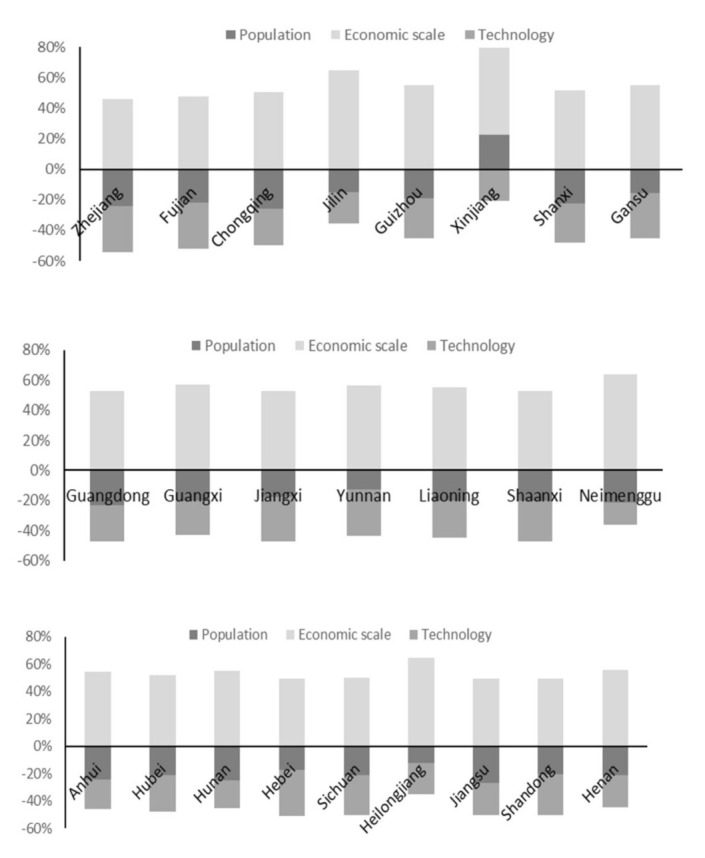
The contribution rate of various effects on carbon emissions from planting industry.

A change of efficiency led to a reduction in planting demand which, in turn, led to a change in carbon emissions from the planting industry. Furthermore, we obtained the contribution rates of the three effects; the results are shown in Figure 3. In general, the contribution of scale effect (except for Shanghai) to carbon emissions of the planting industry in all 31 provinces was positive, indicating that the expansion of agricultural economic scale had a positive driving effect on the carbon emissions of the planting industry. Technological and population effects (except for in Tibet and Xinjiang) had a negative influence on the carbon emissions of the planting industry, such that enhancing the efficiency of the technology and population growth inhibited planting industrial carbon emissions; however, this inhibition was less than the positive scale effect on the carbon emissions. Thus, if enhancing the efficiency of technology and the population growth potential effects on the planting industry have not been fully realized, the rebound effect of enhancing the efficiency of technology and population growth within a certain period may be limited and may not effectively control the excessive growth of the carbon emissions from the planting industry. The effects of technology, population, and scale on the carbon emissions of the planting industry should be considered.

**Figure 4:**
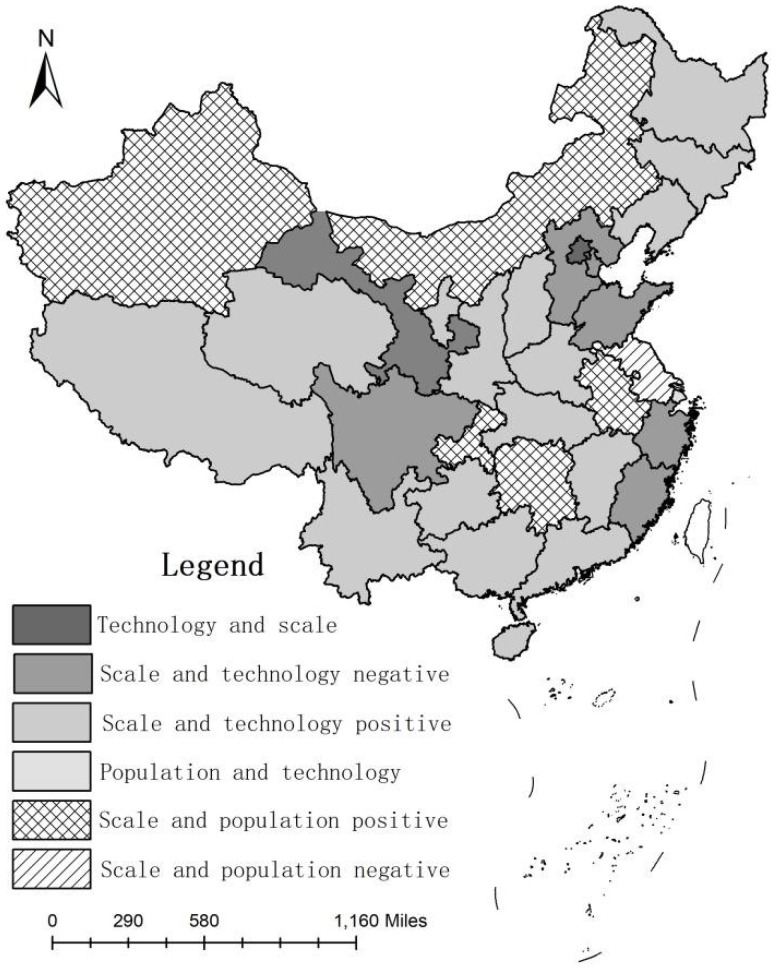
The spatial difference of main factors of carbon emissions from the planting industry.

Figure 4 shows that each province had a significant effect on the contribution of the planting industry carbon emissions. In Hebei, Tianjin, Shandong, Zhejiang, Fujian, Sichuan, and Gansu, the scale and technology effects were the main factors affecting planting industry carbon emissions; however, the process of planting industry carbon emissions reduction by the inhibitory effect of population growth and the improvement of technical efficiency was significant, such that these areas should focus on agricultural economic development processes which are closely integrated with science and technology. This is key to addressing the sustainable development and utilization of agricultural resources; that is, through the improvement of technical efficiency to reduce carbon emissions from planting industrial technology. In Shanghai, the contributions of the population and technology effects to carbon emissions of the planting industry were higher than that of the scale effect, such that the negative driving effect of technological progress and population growth on the carbon emissions of the planting industry were more significant. Therefore, we should optimize the quality of the population and focus on improving the scientific and technological quality of agricultural workers. At the same time, it is necessary to accelerate the formation of new drivers of agricultural economic development, as led and supported by agricultural science and technological innovation, and to promote the transformation of agricultural industry development into an innovation-led type. We should reasonably regulate the growth rate and development scale of the agricultural economy, optimize the allocation of agricultural resources, and truly “decouple” the growth of the agricultural economy from the carbon emissions of the planting industry.

### 4.5. Discussion

(1) Due to the regional characteristics of agricultural resources and the great differences in the level of economic development between regions, carbon emissions from the planting industry were unbalanced. The development of the agricultural economy is highly dependent on resource endowment, which eventually leads to differences in the degree of carbon emissions between agricultural economic development and the planting industry in different provinces. In this paper, we analyzed the agricultural economic growth in 31 Chinese provinces, along with the degree of planting industry carbon emissions, to assess the decoupling relationship and spatial distribution patterns, as well as the formation of a spatial pattern of exploration on the internal mechanism, in order to discern specific factors to contribute to the development of effective countermeasures and to make the research question more targeted, which provides a scientific basis for the sustainable development of China’s agricultural economy.

(2) Based on the agricultural economic growth and planting industry carbon emissions decoupling analysis, China’s agricultural economic development mostly achieved a strong decoupling state with carbon emissions; however, with significant agricultural provinces slowing in agricultural economic growth, to prevent soaring planting industry carbon emissions, agricultural economic growth, the path of the planting industry and its carbon emissions, and their decoupling relationship still need further study and contemplation. $\left(A\right)$ The mode of economic growth and control resource consumption should be changed, in order to achieve the goal of absolute decoupling between economic development and carbon emissions of the planting industry. At present, China is in the process of agricultural modernization, and economic development cannot be achieved without the support of infrastructure and national policies. In order to truly achieve sustainable development, on one hand, it is necessary to optimize the allocation of agricultural resources and regulate the basic raw materials with high energy consumption and high pollution. On the other hand, it is necessary to change the mode of economic development, optimize and upgrade the agricultural industry, and give priority to the development of the green agricultural industry with low emissions, low energy consumption, high technological content, and high added-value. $\left(B\right)\;$The economic growth rate should be reasonably regulated, and the efficiency of resource utilization should be improved. At the present level of technological development, the reduction in carbon emissions from the planting industry brought by the improvement of technical efficiency has been very limited. In order to truly achieve emission reductions and sustainable development, we must optimize the quality of the population, further strengthen scientific and technological innovation and management, reduce resource consumption and pollutant emission technologies, and make full use of resources. More importantly, it is necessary to properly regulate the economic growth rate and scale of expansion, in order to prevent the unnecessary waste of resources. $\left(C\right)\;$The provinces should be assessed, based on regional function orientation and marine resource endowment. The differentiation of the agricultural industry development strategy in Guangdong, Shandong, Fujian, Zhejiang, Jiangsu, Tianjin, Liaoning, and Hebei should focus on the transformation of the mode of economic growth, powered by scientific and technological innovation, thus promoting agricultural development technology and facilitating agricultural economic growth while further reducing planting industry carbon emissions. On the other hand, Shanghai should rely on talent, technological, and location advantages to accelerate the transformation and upgrading of the agricultural industry, actively cultivating strategic emerging industries (e.g., smart agriculture and ecological agriculture), and maintain strong decoupling between agricultural economic growth and carbon emissions from the planting industry. Guangxi and Hainan should further optimize their agricultural industrial structure, speed up the green transformation of agriculture, appropriately reduce high-pollution planting, actively utilize their coastal regional advantages, explore and develop the intensive and deep processing of aquatic products, and realize the efficient utilization of agricultural resources. Heilongjiang, Sichuan, Hunan, and other major agricultural provinces should maintain the advantages of traditional agricultural bases, strengthen industrial scientific and technological innovation through policy guidance, build agricultural science and technology parks and other forms of scientific and technological innovation platforms, and fully release the supply potential of their superior agricultural resources.

## 5. Conclusions

(1) We used the IPCC method to calculate economic development and carbon emissions in China. In 1978 (at the same time as the base year), we calculated the agricultural real GDP; the results showed that, in the period 1998–2019, an average annual rate of 6.8% GDP growth was observed, driving carbon emissions by an average of 14.27%, along with an increased dependence on resources and the environment to facilitate economic growth. This indicates that economic growth had a positive correlation with planting industry carbon emissions, but also illustrates that the current rural development in China is still in the high resource consumption, high energy consumption, high emission, and high pollution extensive development pattern. However, after 2016, carbon emissions from the planting industry showed a downward trend and the change tended to be stable, while the agricultural economy grew rapidly. The growth rate of carbon emissions from the planting industry was significantly lower than that of the agricultural economy, indicating that, with the growth of the agricultural economy, the pressure on resources and the environment posed by China’s agricultural industries showed a slowing trend.

(2) Using the Tapio decoupling model, it was found that, in 2019, the regions with significant decoupling showed a trend of spatial agglomeration. Except for Heilongjiang, Sichuan, Hunan, and other major agricultural provinces, the gap of regional decoupling degree has gradually narrowed. It is worth noting that the input data relating to chemical fertilizer, pesticide, diesel, and other resource factor use have increased year by year, which indicates that China currently pays too much attention to the control of environmental pollution at the end and has neglected research on the resource management mechanism at the source, which is not conducive to reducing pressure on agricultural resources and the environment.

(3) An empirical analysis of the main driving mechanisms of carbon emissions from the planting industry in the process of agricultural economic growth in China by using a complete decomposition model showed that the carbon emissions of the planting industry in China were subject to the driving effect of economic scale expansion and the restraining effects of population growth and technology. The economical scale effect was the main reason for the large increase in carbon emissions from the planting industry, which was greater than the effect of technical efficiency on the decrease in carbon emissions from the planting industry. The improvement of resource utilization and progress in environmental treatment technology are the main reasons for the decrease in carbon emissions from the planting industry. In Tianjin, Hebei, Liaoning, Jiangsu, Fujian, Shandong, and Guangdong, the main factor affecting carbon emissions from the planting industry was the technology effect; moreover, in Shanghai, Zhejiang, Guangxi, and Hainan, the main factor affecting carbon emissions from the planting industry was the population effect. In this regard, focusing on improving technical efficiency, appropriately controlling the population scale, rationally regulating the scale of agricultural economic development, and optimizing the agricultural industrial structure are key to achieving “decoupling” between agricultural economic development and the carbon emissions of the planting industry.

(4) In this manuscript, the carbon emissions from farming industry evaluation remains a preliminary study. There are a few problems that have yet to be researched. Firstly, the selection criteria of the different indicators and sources and coefficients of carbon emissions would produce different results, and further affect decoupling analyses. In the future, there is still a need to use a variety of other indicators, evaluation standard and methods, for analysis of the existing robustness, and a reliability of inspection. Secondly, the Tapio method is essentially “data driven”, mainly by the data statistical description, and lacks the theory of the explanatory power of the model. Considering the effect of space, exploratory spatial data analysis (ESDA)—confirming the space data analysis method—could be a further interpretation of regional water scarcity differences.

## Figures and Tables

**Table 1:** Sources and coefficients of carbon emissions in the planting industry.

Source of Carbon	Discharge Coefficient	Reference Source
Fertilizer	0.8956 kg/kg	Oak Ridge National Laboratory
Pesticide	4.9341 kg/kg	Oak Ridge National Laboratory
Agricultural film	5.1800 kg/kg	Nanjing Agricultural University
Agricultural diesel	0.5927 kg/kg	Intergovernmental Panel on Climate Change
Agricultural planting	3.1260 kg/hm^2^	China Agricultural University
Agricultural irrigation	25 kg/hm^2^	Intergovernmental Panel on Climate Change

**Table 2:** Judgment standard of decoupling model between the agricultural gross domestic product (AED) and carbon emissions of the planting industry (CEP).

State	△CEP	△AED	β	Description
Strong decoupling	<0	>0	β < 0	Economic development, carbon decline
Weak decoupling	>0	>0	0 < β ≤ 1	Economic development, carbon grow slowly
Recessive decoupling	<0	<0	β > 1	Economy slows down, carbon drops quickly
Extended negative decoupling	>0	>0	β > 1	Economy is growing slowly, carbon grows quickly
Weak negative decoupling	<0	<0	β	Economic recession, carbon slowing down
Strong negative decoupling	>0	<0	β < 0	Economic recession, carbon grow

**Table 3:** The result of ADF unit root test.

	DW	ADF	Critical Value is 1%	Critical Value is 5%	Smooth?
LAED	1.84	−2.579	−4.053	−3.742	No
LCEP	2.13	−2.469	−4.053	−3.742	No
iLAED	2.06	−1.976	−1.639	−1.085	Yes
iLCEP	2.22	−1.432	−1.263	−1.369	Yes

**Table 4:** The Granger test of the logarithmic treatments of AED and CEP (LAEG and LCEP, respectively).

Null Hypothesis	Statistics F	Probability Value
LAED is not the cause of LCEP	3.682	0.013
LCEP is not the cause of LAED	0.054	0.765

**Table 5:** The decoupling relationships from 1998 to 2019.

Decoupling	1999	2003	2007	2011	2015	2019
Beijing	−0.573	−1.362	−1.547	−2.852	2.060	2.406
Tianjin	40.964	−0.180	1.061	−0.658	−0.551	−4.665
Hebei	0.155	−0.283	0.287	0.166	−1.559	−0.939
Shanxi	0.022	−0.412	−0.048	0.549	−6.202	−0.766
Neimenggu	0.741	−0.302	2.322	0.125	2.410	0.379
Liaoning	0.008	−0.073	0.788	−0.010	1.800	0.408
Jilin	0.210	−0.014	1.993	1.004	0.476	−0.259
Heilongjiang	−0.177	−2.356	3.593	0.660	2.148	−0.212
Shanghai	4.420	−2.683	−0.431	1.070	−1.471	1.505
Jiangsu	−0.086	12.640	0.401	−0.099	2.363	−1.276
Zhejiang	0.198	−1.289	−2.172	−0.103	0.988	−1.098
Anhui	−0.031	−0.303	0.468	0.425	0.842	−4.248
Fujian	0.633	−0.652	−0.569	0.017	−0.391	−1.536
Jiangxi	0.607	−0.906	0.418	0.063	1.292	−0.702
Shandong	1.229	−0.287	0.384	0.039	−1.379	4.491
Henan	0.343	0.328	0.962	0.638	−0.497	−8.910
Hubei	−1.806	−0.167	0.236	0.292	1.310	−4.551
Hunan	0.313	0.287	−0.138	0.396	1.711	−0.094
Guangdong	−0.022	−0.635	0.047	0.558	3.339	−3.732
Guangxi	−0.046	−1.217	0.080	0.389	3.290	−0.425
Hainan	−0.554	0.963	0.502	0.404	1.516	−1.812
Chongqing	−2.290	−0.750	−0.280	0.464	−0.198	−0.127
Sichuan	0.330	−0.272	0.388	0.324	−2.331	0.453
Guizhou	0.965	−0.347	−1.025	4.281	−2.700	0.943
Yunnan	−1.067	0.089	0.032	0.872	−1.726	−0.616
Xizang	0.526	−1.732	0.574	0.528	−1.093	−0.223
Shanxi	−1.670	−0.021	0.228	0.448	−0.190	−0.219
Gansu	−2.297	−0.679	1.610	1.050	−0.187	−0.704
Qinghai	−1.828	−2.369	1.890	0.469	3.254	−0.020
Ningxia	0.528	−1.692	1.075	0.366	1.457	0.007
Xinjiang	−1.545	−0.333	0.609	1.030	1.091	4.126

## Data Availability

Data are available on request due to restrictions, e.g., privacy or ethical. The data presented in this study are available on request from the corresponding author. The data are not publicly available due to the strict management of various data and technical resources within the research teams.

## Appendix A

**Table A1:** Carbon emissions (10^4^t) from the planting industry in rural China from 1998–2019.

Regions	1998	1999	2000	2001	2002	2003	2004	2005	2006
Beijing	43.87	43.19	39.49	34.18	29.26	27.83	28.23	28.64	29.48
Tianjin	40.28	41.93	39.37	39.80	39.34	38.91	44.24	45.55	46.58
Hebei	637.95	642.21	633.69	629.89	637.92	626.93	642.14	668.54	668.28
Shanxi	228.98	228.16	227.02	215.91	227.33	220.86	228.74	235.76	243.79
Neimenggu	295.80	297.99	280.56	280.91	304.08	298.67	317.93	338.74	354.60
Liaoning	263.11	263.22	252.08	262.04	263.28	261.89	272.75	280.80	285.97
Jilin	261.18	261.95	270.05	288.41	297.52	297.27	343.08	325.84	338.26
Heilongjiang	442.08	439.73	428.61	450.87	481.84	454.59	490.84	506.66	541.43
Shanghai	43.08	47.08	42.02	40.96	36.47	34.22	32.19	32.42	31.58
Jiangsu	649.97	647.38	631.27	621.94	618.22	610.41	618.17	631.26	637.81
Zhejiang	246.86	248.48	231.33	221.83	214.97	205.00	209.91	214.65	215.80
Anhui	551.05	549.69	558.45	571.86	579.50	590.72	597.09	612.94	625.20
Fujian	223.93	232.02	223.74	216.64	218.83	214.12	215.33	216.57	216.59
Jiangxi	291.03	301.64	289.34	285.91	289.61	282.52	311.61	324.45	330.39
Shandong	854.24	903.58	859.81	866.86	886.86	872.60	882.08	916.14	947.71
Henan	809.64	829.64	849.75	865.65	911.95	904.48	933.20	966.01	997.77
Hubei	511.17	493.63	479.62	472.49	477.85	473.22	496.41	505.64	527.23
Hunan	445.56	450.17	449.05	445.18	443.73	448.31	479.01	490.34	495.29
Guangdong	352.05	351.75	350.97	364.37	358.34	353.33	355.80	359.46	368.59
Guangxi	322.56	321.44	324.28	335.05	359.53	342.03	358.81	366.55	371.27
Hainan	48.06	45.19	52.03	51.61	55.35	60.15	65.76	62.52	67.34
Chongqing	186.58	184.87	183.71	181.91	182.86	176.83	182.91	184.63	185.52
Sichuan	525.78	534.45	524.78	517.39	517.29	510.12	520.72	531.15	541.12
Guizhou	180.37	186.28	190.29	188.30	196.90	193.76	194.78	199.00	204.50
Yunnan	280.85	267.36	301.94	313.30	319.10	320.66	333.03	343.57	353.53
Xizang	12.88	13.24	13.03	13.41	14.22	13.38	14.00	14.39	14.37
Shaanxi	285.99	296.97	287.53	277.01	272.37	272.13	274.63	283.15	287.36
Gansu	203.99	206.34	201.24	199.31	206.26	198.56	205.07	212.20	214.79
Qinghai	24.31	23.84	22.99	22.68	22.07	20.06	19.67	20.01	20.70
Ningxia	63.97	65.42	59.31	59.54	64.57	61.95	63.64	65.51	68.22
Xinjiang	257.21	246.48	245.82	254.66	265.88	258.62	272.10	289.74	310.29

**Table A2:** Carbon emissions (10^4^t) from the planting industry in rural China from 1998–2019 (continued Table A1).

Region	2006	2007	2008	2009	2010	2011	2012
Beijing	29.48	28.48	30.45	29.69	29.34	28.59	27.77
Tianjin	46.58	47.27	47.41	47.59	47.10	45.92	46.18
Hebei	668.28	675.95	677.27	683.56	690.89	695.72	701.38
Shanxi	243.79	243.81	251.20	256.16	263.69	272.52	278.22
Neimenggu	354.60	386.71	410.92	436.79	450.88	454.21	473.49
Liaoning	285.97	294.99	294.26	302.02	321.04	320.84	329.51
Jilin	338.26	346.35	359.28	371.61	383.25	402.87	420.76
Heilongjiang	541.43	621.18	635.95	674.42	708.28	737.27	763.64
Shanghai	31.58	31.31	32.26	29.94	28.48	28.26	26.93
Jiangsu	637.81	645.75	639.36	642.82	640.54	637.99	635.71
Zhejiang	215.80	205.02	207.96	209.96	208.45	207.68	208.48
Anhui	625.20	635.73	649.99	657.58	668.86	680.23	686.50
Fujian	216.59	211.79	212.14	215.09	216.03	216.19	215.70
Jiangxi	330.39	336.06	343.27	347.91	356.07	357.01	361.01
Shandong	947.71	962.26	876.13	938.95	942.93	944.39	948.49
Henan	997.77	1034.26	1071.20	1103.49	1133.20	1159.96	1174.88
Hubei	527.23	533.08	565.58	582.17	595.57	603.22	607.75
Hunan	495.29	492.56	500.50	517.08	524.43	533.16	542.86
Guangdong	368.59	369.15	392.08	401.55	405.54	415.04	419.33
Guangxi	371.27	372.91	376.68	387.70	396.72	404.12	412.01
Hainan	67.34	70.05	80.38	89.27	89.36	91.60	87.06
Chongqing	185.52	180.59	186.29	191.00	191.89	196.43	196.79
Sichuan	541.12	548.48	554.42	562.27	564.82	573.06	578.23
Guizhou	204.50	197.58	208.08	216.04	222.05	233.45	240.19
Yunnan	353.53	354.16	372.24	382.15	401.19	422.19	442.66
Xizang	14.37	14.71	16.44	16.77	17.11	17.42	17.90
Shaanxi	287.36	290.63	297.72	314.00	327.15	335.80	365.64
Gansu	214.79	228.62	238.06	245.99	260.03	276.14	289.36
Qinghai	20.70	22.42	24.02	24.52	25.82	26.41	27.31
Ningxia	68.22	72.99	73.25	74.92	78.76	80.23	81.16
Xinjiang	310.29	323.70	365.66	378.75	397.47	424.08	440.33

**Table A3:** Carbon emissions (10^4^t) from the planting industry in rural China from 1998–2019 (continued Table A2).

Regions	2013	2014	2015	2016	2017	2018	2019
Beijing	24.32	20.67	16.07	15.41	13.70	12.54	11.01
Tianjin	45.21	43.87	43.26	41.57	37.92	35.92	32.83
Hebei	697.43	681.51	653.89	667.73	656.08	620.00	600.21
Shanxi	282.42	282.55	265.03	262.94	257.91	252.60	247.48
Neimenggu	485.83	493.38	529.60	541.27	545.36	556.80	563.33
Liaoning	328.64	324.60	346.80	343.03	343.18	357.08	361.81
Jilin	426.40	434.65	444.58	461.55	460.93	452.52	449.31
Heilongjiang	786.29	776.56	862.66	901.67	893.71	884.38	875.97
Shanghai	25.28	24.34	29.21	26.91	25.62	24.78	23.65
Jiangsu	628.46	597.31	643.31	638.95	628.19	629.24	622.41
Zhejiang	207.00	200.73	203.64	199.22	195.77	188.16	183.32
Anhui	710.49	689.72	714.37	729.23	719.85	709.07	699.22
Fujian	220.19	215.96	212.83	219.02	209.20	197.65	187.25
Jiangxi	361.66	354.54	372.41	372.26	372.41	357.34	352.41
Shandong	937.81	927.44	874.29	883.01	861.70	845.85	826.36
Henan	1183.97	1184.36	1158.47	1180.74	1167.90	1162.73	1152.44
Hubei	608.77	600.48	636.24	638.10	623.41	604.09	587.86
Hunan	553.69	542.66	576.16	593.06	595.97	594.70	596.22
Guangdong	412.77	416.37	462.40	476.52	475.53	428.53	412.21
Guangxi	421.10	420.19	474.11	497.34	498.94	490.49	488.74
Hainan	90.88	88.43	95.66	93.75	91.86	80.42	75.35
Chongqing	196.55	190.71	188.93	194.82	194.50	192.32	191.37
Sichuan	575.67	562.59	514.07	536.95	538.07	522.12	517.55
Guizhou	234.93	236.29	194.82	217.19	235.25	238.63	251.79
Yunnan	455.12	461.91	414.86	426.93	429.14	414.53	411.14
Xizang	18.61	18.60	17.84	18.73	18.59	18.42	18.27
Shaanxi	365.39	348.70	345.32	348.76	348.52	347.65	347.20
Gansu	296.11	302.97	299.92	307.01	298.69	286.08	276.33
Qinghai	26.67	25.87	30.16	30.51	30.97	29.97	29.95
Ningxia	81.92	80.12	85.46	86.28	87.49	85.14	85.16
Xinjiang	482.06	543.88	578.88	587.02	580.03	584.23	580.97

## Appendix B

**Table A4:** The influence of various effects on carbon emissions (10^4^t) from the planting industry in rural China.

Regions	Population Effects	Scale Effects	Technology Effects	Rebound Effects
Beijing	−6.265	6.459	−33.053	−32.859
Tianjin	−17.851	48.458	−38.055	−7.449
Hebei	−401.858	1120.468	−756.352	−37.742
Shanxi	−112.013	260.243	−129.737	18.493
Neimenggu	−204.867	611.170	−138.769	267.534
Liaoning	−194.130	527.916	−235.085	98.701
Jilin	−95.778	415.384	−131.473	188.134
Heilongjiang	−175.173	949.939	−340.874	433.892
Shanghai	−8.824	−4.403	−6.209	−19.437
Jiangsu	−651.068	1193.656	−570.144	−27.556
Zhejiang	−190.919	362.302	−234.930	−63.547
Anhui	−429.642	945.938	−368.133	148.163
Fujian	−190.295	417.151	−263.539	−36.683
Jiangxi	−189.329	563.451	−312.737	61.385
Shandong	−643.402	1541.795	−926.274	−27.881
Henan	−667.538	1744.798	−734.462	342.798
Hubei	−370.907	920.797	−473.197	76.693
Hunan	−378.837	840.639	−311.144	150.658
Guangdong	−246.706	564.582	−257.711	60.164
Guangxi	−244.460	682.933	−272.297	166.176
Hainan	−17.368	122.797	−78.143	27.287
Chongqing	−227.184	445.055	−213.075	4.796
Sichuan	−387.224	917.956	−538.958	−8.227
Guizhou	−135.519	389.244	−182.300	71.424
Yunnan	−133.430	581.238	−317.521	130.287
Xizang	1.923	11.139	−7.671	5.390
Shaanxi	−230.110	599.706	−308.380	61.217
Gansu	−117.048	417.564	−228.178	72.339
Qinghai	−6.182	31.861	−20.040	5.639
Ningxia	−27.116	111.023	−62.715	21.192
Xinjiang	123.927	314.390	−114.565	323.753
